# Autoimmune Disease in Women: Endocrine Transition and Risk Across the Lifespan

**DOI:** 10.3389/fendo.2019.00265

**Published:** 2019-04-29

**Authors:** Maunil K. Desai, Roberta Diaz Brinton

**Affiliations:** ^1^School of Pharmacy, University of Southern California, Los Angeles, CA, United States; ^2^Center for Innovation in Brain Science, University of Arizona, Tucson, AZ, United States; ^3^Departments of Pharmacology and Neurology, College of Medicine, University of Arizona, Tucson, AZ, United States

**Keywords:** women, autoimmne disease, menopause, puberty, gender differences

## Abstract

Women have a higher incidence and prevalence of autoimmune diseases than men, and 85% or more patients of multiple autoimmune diseases are female. Women undergo sweeping endocrinological changes at least twice during their lifetime, puberty and menopause, with many women undergoing an additional transition: pregnancy, which may or may not be accompanied by breastfeeding. These endocrinological transitions exert significant effects on the immune system due to interactions between the hormonal milieu, innate, and adaptive immune systems as well as pro- and anti-inflammatory cytokines, and thereby modulate the susceptibility of women to autoimmune diseases. Conversely, pre-existing autoimmune diseases themselves impact endocrine transitions. Concentration-dependent effects of estrogen on the immune system; the role of progesterone, androgens, leptin, oxytocin, and prolactin; and the interplay between Th1 and Th2 immune responses together maintain a delicate balance between host defense, immunological tolerance and autoimmunity. In this review, multiple autoimmune diseases have been analyzed in the context of each of the three endocrinological transitions in women. We provide evidence from human epidemiological data and animal studies that endocrine transitions exert profound impact on the development of autoimmune diseases in women through complex mechanisms. Greater understanding of endocrine transitions and their role in autoimmune diseases could aid in prediction, prevention, and cures of these debilitating diseases in women.

## Key Points

- Women undergo three major endocrinological transitions: puberty, pregnancy and menopause.
- These endocrine transitions exert a significant influence on the innate and adaptive immune system due to the interaction between the hormonal milieu, innate and adaptive immune system as well as pro- and anti-inflammatory cytokines.
- Concentration-dependent effects of estrogen on the immune system, the role of progesterone, androgens, leptin, oxytocin and prolactin; and the interplay between Th1 and Th2 immune responses together maintain a delicate balance between defense against pathogens, immunological tolerance, and autoimmunity.
- Human epidemiological data, animal studies, and mechanistic experiments have demonstrated a strong link between endocrine transition states in women and development of certain autoimmune diseases such as multiple sclerosis, systemic lupus erythematosus, type 1 diabetes mellitus, rheumatoid arthritis, and psoriasis.
- Greater understanding of endocrine transitions and their role in autoimmune diseases could aid in prediction, prevention, and cures of these debilitating diseases in women.

## Endocrine Transitions in Women Increase their Susceptibility to Autoimmune Diseases

### Introduction

Autoimmune (AI) diseases include over 80 disorders and are the third most common category of disease in the United States after cancer and cardiovascular disease, affecting ~5 to 8% of the population or 14.7 to 23.5 million people (1, 2). While the clinical manifestations of AI diseases vary in terms of affected tissues, age of onset, and response to immunosuppressive treatments, a shared feature of all AI diseases is the contribution of host immune-mediated responses to tissue injury (3). AI diseases are the result of a complex interaction between genetic and environmental factors, and most of these factors have not yet been definitively identified (4).

**Figure 1:**
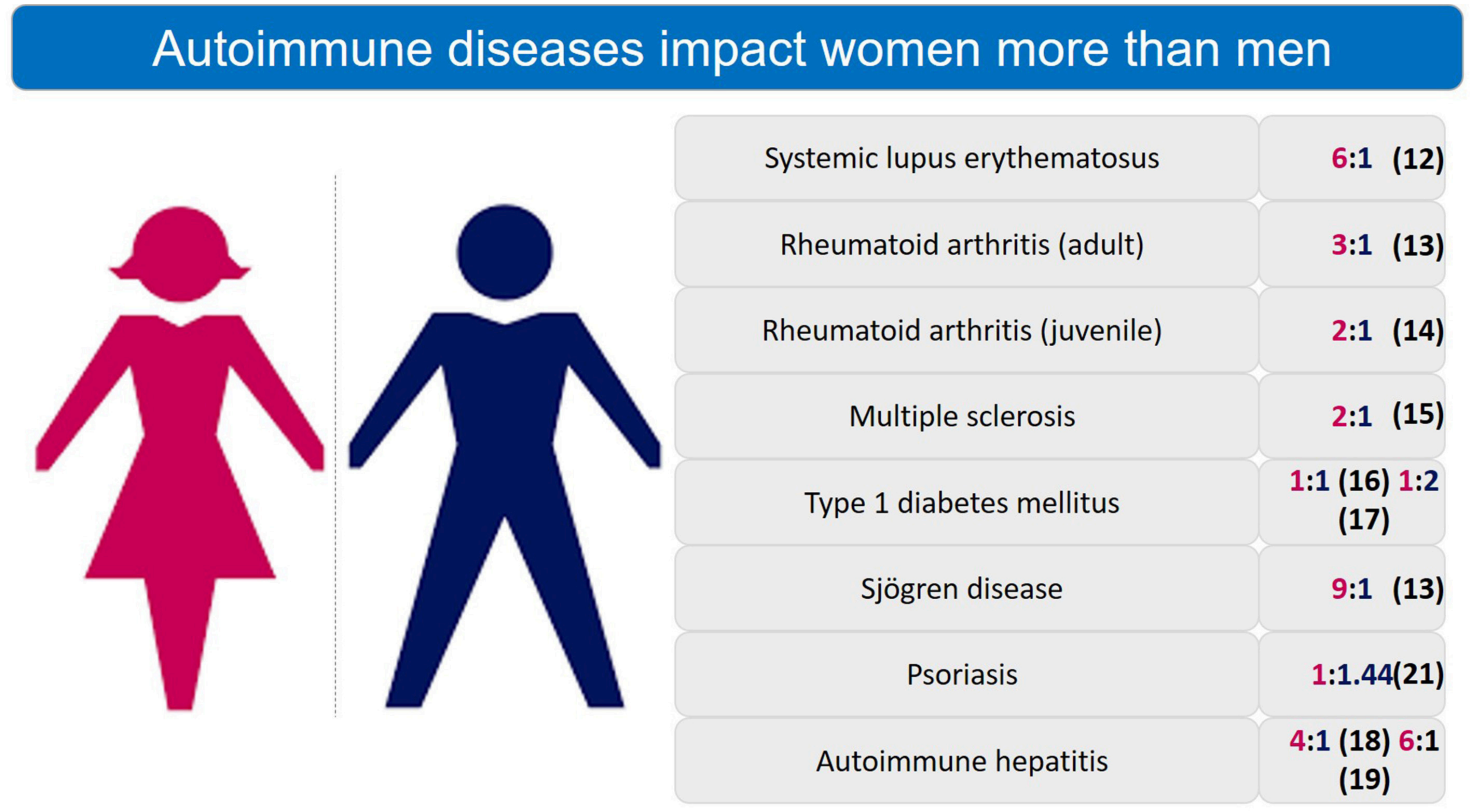
Sex differences in prevalence ratio of autoimmune diseases. Autoimmune diseases are significantly more prevalent in women than men.

Women constitute ~78% of those affected by autoimmune diseases, bearing a disproportionate burden of the high morbidity associated with these chronic conditions (5–8). Female sex is a risk factor for polyautoimmunity (9). The “gender gap” in autoimmunity has been well known for over 20 years (10, 11) and AI diseases are a leading cause of death among young and middle-aged women (12). Figure 1 enumerates the gender differences in prevalence of autoimmune diseases (8, 13–20).

AI diseases have a strong gender-specific component with female hormones playing a major immunomodulatory role that depends on their concentration in the bloodstream, the concomitant levels of other hormones, and the age of the host (21). Commencing at puberty, women undergo cyclical hormonal changes until menopause, called menstrual cycle which makes pregnancy possible (22, 23). Pregnancy and post-gestational phases are characterized by hormonal fluctuations that have a long-lasting holistic impact on women's health (24). Menopause is a state of reproductive senescence when menses cease and is preceded by a 3–4 year period of irregular reproductive function known as the perimenopausal transition stage (25). In a rat model, which mimics both endocrine and chronological aging observed in women (26), perimenopause was characterized by a decline in expression of genes involved in bioenergetics, as well as differential regulation of the inflammatory response (26). There is evidence that the changing hormonal milieu during the menopausal transition, and its effect on inflammatory processes plays a role in the increased susceptibility of peri- and post-menopausal women to autoimmune diseases (Table 1) (27–29). For example, in women aged around 50 years, neutrophil percentage dropped whilst lymphocyte percentage rose (30), subjecting perimenopausal women to increased risk of lymphocyte mediated autoimmune diseases.

**Table 1:** Autoimmune diseases and transition states.

	**Puberty**	**Pregnancy**	**Menopause**
T1DM	•Peak incidence between 5 and 7 years and at puberty. •Greater risk of complications in girls.	•Worsens pregnancy outcome vs. T2DM. •Thyroid antibodies associated with Gestational DM but lack predictive value. •High TSH and thyroid autoimmunity increase GDM risk.	•Females with T1Dm have higher mortality than men. •Microvascular complications in T1DM result in pre-mature menopause. •Women bear unequal burden of sequelae of T1DM vs. men.
SLE	•Increase incidence in girls post-puberty. •ANA titer higher in female children. •Effect on height higher in males. •Menarche pushed to higher age.	•Lower E2 levels in pregnant lupus patients vs. pregnant controls. •Prolactin levels positively associated with severity. •Flares more common with increase in estrogen.	•Decreased frequency of flares after menopause but more damage from each flare. •Sexually dimorphic immune response in the gut mucosa of males and females.
RA	•Earlier age at menarche increases risk.	•Pregnancy and breast feeding is protective.	•Irregular menstrual cycle increases risk. •Early age at menopause significantly associated with RA. •More joint destruction in post-menopausal women. •HRT protective.
MS	•Sex ratio rises to 2.2:1 post-puberty. •Late menarche decreases risk (females-only). •Pre-pubertal ovariectomy decreases risk. •Spike in relapse during peri-menarche and incidence peak post-menarche. •Higher leptin in females may increase risk.	•Relapse rate reduced during pregnancy but worsens after delivery. •Breast-feeding reduces relapse.	•Peak incidence in perimenopausal age group. •Post-menopausal women have worse symptoms and higher severity. •More frequently primary progressive MS than relapsing-remitting MS in post-menopausal age group.
Psoriasis	•Perimenarchal increase in incidence.	•Decreased severity during pregnancy. •Higher estrogen and higher E2 to progesterone ratio results in improvement.	•Post-menopausal exacerbation of psoriasis. •Late onset psoriasis is more common in women than in men.

The dynamic hormonal milieu during these transitions is significant from the perspective of sexual and reproductive health, but also influences other aspects of female health, such as susceptibility to infection and autoimmunity, tissue integrity, ophthalmology, movement, cognition, disability, and overall quality of life. A better understanding of these transitions and their association with autoimmune diseases represents a critical component of women's health.

### Autoimmune Diseases and Endocrine Transition States

Below five relatively common autoimmune diseases, namely Multiple Sclerosis, Systemic Lupus Erythematosus, Type 1 Diabetes Mellitus, Rheumatoid Arthritis, and Psoriasis, have been discussed in the context of endocrinological transition states in women: puberty, pregnancy, and menopause.

#### Multiple Sclerosis

Multiple sclerosis (MS) is largely prevalent among women of reproductive age and is less common in men, with twice as many women affected as men (31). However, disease progression and neurodegeneration occur more rapidly in men (32). MS is closely linked to the female reproductive and hormonal cycle, and the majority of cases of MS in adolescence are observed post-puberty (33).

Over the past six decades the absolute number of cases of MS has increased with the increase in the female-to-male sex ratio among patients. The sex ratio among MS patients was reported to be 1.4 in 1955 (34), reaching a 2:1 ratio in the 1980s (35), approaching a ratio of 3:1 in mid-2000s (36, 37) and then surpassing it in recent years (38–41). MS after 50 years of age is greater in women and exhibit a more rapidly degenerating phenotype. Postmenopausal status has been associated with increased disease severity, even adjusting for disease duration and type, and earlier age at menopause also appeared to have a strong correlation with increased disease severity (42).

##### Puberty

Pediatric MS, defined as MS diagnosis at <8 years of age has been observed in ~3–5% of MS patients (43–45), while MS onset after the age of 50 years (known as Late-Onset MS or LOMS) occurs in ~5% of patients (46). Pre-pubertal MS onset is uncommon (47, 48), however, the incidence of MS increases after puberty (45, 47–49). The sex ratio of MS incidence is approximately equal in children <13 years of age (pre-pubertal) but increases to 2.2:1 with double the number of females affected in the 13–18 years age group (45, 47, 48). Acute demyelinating disorders (ADS) such as transverse myelitis (50) and acute disseminated encephalomyelitis (51) are more common among children <10 years of age and show no female preponderance. However, in women, chronic demyelinating diseases, such as MS and neuromyelitis optica, are more frequent post-puberty than in men (33, 45, 47, 48, 50, 51). Ahn et al. (52), reported that female children with ADS and a later age of menarche were less likely to be diagnosed with MS. MS patients showed a spike in relapse risk only during the peri-menarche transition, and a peak in disease onset was observed 2 years after menarche (53). Earlier age at menarche has been shown to increase the risk of MS in female subjects significantly (*p*-value = 0.00017), while in male subjects the association between the risk of MS and earlier age at puberty was not found to be significant (54). Clinical and epidemiological observations have led to the conclusion that puberty increases the risk of MS, with post-pubertal females more likely to develop MS than post-pubertal males of the same age group.

##### Pregnancy

A systematic review (55) of 22 publications confirms the reduction of relapses during pregnancy, due to increased levels of sex hormones produced (56). MS symptoms also vary during the menstrual cycle (57). Pregnancy has a protective effect on MS. For pregnant MS patients, the relapse frequency of MS was reduced to half during the latter half of pregnancy (18 weeks gestation and beyond), and during the third trimester the relapse rate was found to be lower by 70% than in the pre-pregnancy state (58–63). After delivery, MS worsened, with frequent relapses in the postpartum period with the highest relapse frequency observed to occur within 3 months of delivery (64). In a Danish study (65), childbirth was found to reduce the risk of MS by ~46% for 5 years following the pregnancy and pregnancies terminated early also conferred protective effect on the risk of developing MS, suggesting that temporary immunosuppression during pregnancy served to protect pregnant women from MS in the period immediately following the pregnancy.

##### Menopause

A British study published in 2014 (66) reported a peak incidence of MS in women between the ages of 40 and 55 years (perimenopausal and menopausal age group). The authors concluded that divergent trends in MS incidence in women and men were established around puberty (12–16 years) and the increased MS incidence in women did not converge with the incidence in men until ~60 years of age (post-menopausal).

Disease progression was comparable among men and women with Late Onset MS (LOMS), defined as MS diagnosis in persons over 50 years of age, while in patients with MS onset at a younger age (<40 years), disease progression was more rapid among men than among women, although the disease is more common in women (46). Bove et al. also noted a greater proportion of men in the LOMS cohort (female: male ratio of 1.86) than in the Adult Onset MS (AOMS) cohort (female: male ratio 2.84) cohort (46). This association suggests that female sex may be a risk factor for an earlier presentation of MS, during the reproductive years, or alternatively, that male sex may have a protective effect. Additionally, misdiagnosis of MS in older patients might be a common phenomenon (67) and delays in diagnosis up to 4.7 years have been reported (68, 69), due to unusual presentation of the disease (70).

Common symptoms shared between menopause and MS include fatigue, urologic, affective, cognitive, and vasomotor symptoms (71). Declining estrogen levels during the perimenopausal transition have been associated with immunological alterations comparable to those observed in MS, which include increased secretion of pro-inflammatory cytokines (IL1, IL6, TNF-alpha) and decreased production of anti-inflammatory cytokines (72). Accelerated cognitive decline has been associated with menopause; this is suggested to result from loss of ovarian estrogens leading to impaired brain repair mechanisms and eventual neurodegeneration (26, 71, 73, 74). Therefore, menopause may alter the immune, inflammatory, and neurodegenerative aspects of reproductive aging, independent of the effects of the MS disease process itself and contribute to the misdiagnosis or delayed diagnosis of MS in peri- and post-menopausal women, as described earlier.

In persons with LOMS, it has been observed that the MS disease type at onset was less frequently relapsing remitting (80% for LOMS vs. 95% for AOMS in female patients); the primary progressive course was more frequently rapidly evolving, with fewer relapses or new gadolinium enhancing lesions; and symptoms more frequently involved motor and coordination symptoms and less frequently involved visual symptoms, compared to individuals with AOMS disease (70, 75–78).

#### Systemic Lupus Erythematosus (SLE)

Systemic lupus erythematosus (SLE) affects a significantly greater number of women than men (13, 79–81). The Michigan Lupus Epidemiology and Surveillance Program (81) reported an increased incidence and prevalence of SLE in women across all ethnicities. The female to male ratio of SLE incidence was 6:1 and SLE prevalence was 10:1, and incidence rates were higher in African American female subjects compared to Caucasian female subjects (81). In late childhood, a trend of increased incidence of SLE was observed among African American girls compared to Caucasian girls (81). In the 20–50 year range, an early incidence peak of SLE has been reported in African American women but there were no significant differences in SLE incidence between African American and Caucasian women after the average age of menopause (81), calculated to be 51 years in the US (82). Globally, estrogen levels were shown to be higher in Asian (Japanese) and African (Bantu) women than in Caucasian women (83) which explains the observation that SLE is more prevalent in some ethnic groups, such as Afro-Americans and Asians, since SLE is antibody-mediated and higher estrogen levels favor antibody-mediated Th2 immune response (84).

A Japanese study (85) stratified patients with SLE by age and sex distribution and found that the overall female to male ratio of SLE was 8.2:1. Furthermore, while the prevalence of SLE among women showed two peaks, between ages 35–39 years and ages 55–59 years, no significant age-dependent peaks were observed among men (85). An epidemiological study in an 80% Caucasian population in Minnesota reported that SLE prevalence peaked between the age 40–49 years (perimenopausal) among women and then decreased sharply in the 50–59 years age group while incidence of SLE in women peaks between the ages of 20 and 29 years, and between the ages of 50 and 59 years (79). The incidence of SLE in men remained low until the 60–69 years age group, increasing thereafter, with a peak in men >70 years of age (79). Men >60 years of age have a higher prevalence of SLE compared to younger men (79). A study published in the UK (13) supports the hypothesis that transitional states in women may play a major role in the development of autoimmune diseases such as SLE. This retrospective cohort study, conducted using the CPRD, a longitudinal database of UK general practice records incepted in 1987 and believed to be representative of the UK population, found that the peak SLE incidence rate was observed in 40–49 years age group among women (perimenopausal) and in the 60–69 years age group among men (13), similar to the Minnesota study (79). The incidence rate of SLE in women fell sharply in the 60–69 years group (postmenopausal), suggesting that risk of autoimmune diseases like SLE may decline in the postmenopausal age group (13, 79).

##### Puberty

The female-to-male ratio in SLE has been reported to vary between 2:1 and 6:1 before puberty, 7:1 to 15:1 after puberty, and 3:1 to 8:1 post-menopause (86, 87), suggesting that, in women, the increase in hormone levels during puberty enhances the risk of development of autoimmune states. An epidemiological study from Taiwan (88) noted a substantial increase in the prevalence of juvenile SLE among Taiwanese girls compared to boys of the same age. Prevalence of SLE in girls was 0.65 per 100,000 children at age one, increasing to 6.7 per 100,000 at age seven, and increasing further to 34.6 per 100,000 at age 15 (88). The prevalence figure for boys was almost zero per 100,000 at ages one and seven, increasing to 7.8 per 100,000 at age 15 (88). The 7–15 years age group spans the pre-pubertal and pubertal years for females in Taiwan with the average age of menarche being 12.1 years (89). The multifold jump in juvenile lupus prevalence in female subjects is an indication that endocrinological transition, i.e., puberty, may play an influential role in immunomodulatory function and correlate with increased susceptibility to autoimmune diseases during the peripubertal transition. Titers and prevalence of antinuclear antibody (ANA), a marker for lupus, increased in children through puberty, particularly among girls (90).

Patients suffering from Cutaneous Lupus Erythematosus (CLE), a variant of SLE, demonstrate equal sex distribution with a female to male ratio of 1:1, but the female to male ratio of CLE patients rose to 4.5:1 if the disease onset was at or after the age of 12 (91). Different sex ratios have been reported according to the subtypes of CLE. Acute CLE has the highest female to male ratio (12:1) while chronic CLE had an almost equal ratio of 1.1:1 (91). Above observations suggest a role of puberty in the development of SLE.

Conversely, SLE exerts sexually dimorphic effects young male and female patients. A study exploring the effect of lupus on height found that there was a significant (*p*-value < 0.0001) reduction in the parent-adjusted height z score with time in girls compared to boys (92). The mean menarche age was higher among juvenile patients with SLE than in controls (*p*-value = 0.0008) despite comparable maternal menarche age in both groups (93).

##### Pregnancy

E2 concentrations are abnormally low in pregnant patients with SLE during periods of increased disease activity, compared with pregnant women not suffering from SLE (94, 95). Serum prolactin and disease activity of SLE have been positively associated in multiple studies (96–100). Abnormally high prolactin levels during pregnancy in SLE also positively correlate with disease activity (95, 101). Furthermore, two double-blind, placebo-controlled human studies have shown that suppression of prolactin with bromocriptine, which also increases estradiol concentration (102) reduces SLE disease activity (103–105). Exacerbation of SLE is more common during the pre-menstrual period and pregnancy, during which women experience increased estrogen levels (84). This phenomenon is in contrast with other autoimmune diseases such as MS, psoriasis and T1DM, which are mediated by T lymphocytes, while SLE is a disease mediated by autoantibodies producing-B lymphocytes; and E2, throughout its concentration range, has been shown to stimulate antibody production by B cells (106).

##### Menopause

The female to male incidence ratio of 2.6 is significantly lower in the late onset SLE group (>50 years age) than in the early onset SLE group in which the ratio is 13.2 (107). Furthermore, there is evidence that early age at menarche, oral contraceptive use, early age at menopause, surgical menopause, and postmenopausal use of hormones were associated with an increased risk of SLE (108). Women suffering from SLE and undergoing menopausal transition show a decreased frequency of exacerbations of SLE after menopause, a decreased SLE Disease Activity Index (SLEDAI), but a greater accumulation of damage in the affected organs from each discrete exacerbation in the postmenopausal period (109–112).

#### Type I Diabetes Mellitus (T1DM)

##### Puberty

Unlike other AI diseases such as MS, RA and SLE, the incidence and prevalence of type 1 diabetes is slightly higher in men and boys than in women and girls (113). The epidemiological data in the International Diabetes Federation Atlas 2013 (6th edition) indicate there are ~500,000 known cases of children (0–14 years) with T1DM worldwide and 50–60% of cases are diagnosed before the age of 15 years (114). A study in the Swedish population (18) found that there were no differences between the sexes in the incidence rate of T1DM in children aged between 3 months and 14 years. For both sexes the incidence of type 1 diabetes peaked twice, first between 5 and 7 years of age, and then at or near puberty (115). The incidence of T1DM in subjects aged between 15 and 39 years is twice that in men compared to women (115). There are fewer cases of T1DM in subjects aged 40 years or above, and in this age group the incidence of T1DM is comparable between men and women (115). In contrast to the Swedish study, a Japanese study assessing 77 male and 95 female participants found that although the incidence of T1DM in the prepubertal age group was comparable between the sexes, while it was higher among female subjects in the pubertal age group (116).

While the above observations indicate that globally male children and adults form the majority of T1DM patients, it is important to note that girls and women suffer from morbid sequelae of T1DM more often than males. The development of T1DM during the pubertal transition in girls is associated with a range of conditions. Girls suffering from T1DM are at a greater risk of excessive weight gain and adiposity during puberty, which may exacerbate insulin resistance (117). Adolescent girls with T1DM are more prone to hyperandrogenism or polycystic ovary syndrome phenotype, potentially adding to the cardiovascular risk profile of these patients (117). End Stage Renal Disease (ESRD) is a serious complication of T1DM (118). Among female patients, pubertal onset (onset between 10 and 19 years of age) of T1DM confers the highest risk of development of ESRD compared to prepubertal onset of T1DM (onset <10 years of age) or adult onset of T1DM (onset >20 years of age) (119). Furthermore, female subjects are at a 29% higher risk of developing retinopathy as a complication of T1DM compared to male subjects (120). Young adult women (20–29 years) with T1DM lose the cardiovascular protection that is otherwise seen in the general female population, and exhibit similar rates of ischemic heart disease as those observed in adult men of 18–49 years of age with T1DM (121–123). Increased insulin resistance has been observed among girls at all Tanner stages of pubertal development (124) compared to boys at the same stages, even adjusting for adiposity, Body Mass Index, waist, or hip circumference. In peri-pubertal girls with T1DM, lower sex hormone binding globulin (SHBG) and high free androgen index (FAI) are associated with higher Body Mass Index Standard Deviation Scores (BMI-SDS) and higher total daily insulin per kilogram bodyweight (125, 126).

Therefore, as the evidence above demonstrates, even though there are more boys with T1DM than girls, girls remain more susceptible to the sequelae of T1DM and these sequelae are exacerbated by pubertal onset of T1DM.

##### Pregnancy

Pregnant women with diabetes mellitus are affected by either T1DM, T2DM or gestational diabetes (GDM). Pregnancy outcomes differ depending on the type of diabetes. Pregnant women with T2DM had lower Hemoglobin A1c (marker of long-term control of blood glucose) and lower insulin requirements, lower maternal weight gain, fewer cesarean deliveries, and gestational age at birth was significantly higher than women with T1DM (127). While fetal losses occurred in both T1DM and T2DM groups, intermediate and late term fetal losses were significantly less common among T1DM patients, and T1DM patients had significantly more fetal losses due to congenital anomalies or prematurity compared to T2DM patients (128). Furthermore, T1DM patients have a higher incidence of complications and of poor pregnancy outcomes than those with T2DM (129). These observations show that while poorly controlled hyperglycemia is a primary characteristic of both T1DM and T2DM, pregnancy outcomes are worse among women with pre-existing T1DM.

##### Gestational diabetes

Gestational diabetes is defined as an intolerance to glucose that is first diagnosed or has its onset during pregnancy (130). Close associations have been documented between GDM and immune system. An increase in number and proportion of different subsets of T lymphocytes have been documented in pregnant women suffering from GDM (131–136). Alterations in proportion of regulatory T cell (T_reg_) subpopulations (137) have also been observed in these women along with reduced function of immunosuppressive T_reg_ cells (137).

A recent meta-analysis (138) concluded that there was a significant association between thyroid antibodies and the risk of GDM but thyroid antibodies measured in the first trimester in pregnant women lacked predictive value for the risk of GDM. Furthermore, presence of thyroid antibodies may not increase the risk of GDM in pregnant women who have a normally functioning thyroid gland (138), although higher than normal thyroid-stimulating hormone (TSH) levels (≥2.5 mU/mL) in pregnant women are associated with GDM (139).

Adiposity-induced inflammation in pregnancy (140), and antigenic load of the fetus (141) have both been implicated as causes in the development of GDM but sex steroid may have a role to play as well (142, 143). It was found that first-trimester Sex Hormone Binding Globulin (SHBG) values were inversely associated with an increased risk of the development of GDM that was diagnosed at 26 to 30 weeks of gestation (144); the association was independent of the influence of maternal weight and other important variables that included age, race, and smoking.

Women with autoimmune GDM are more likely than women with non-autoimmune GDM to show pancreatic autoantibody positivity (145). Even though both pregnancy with GDM (146) and healthy pregnancy (147) may be associated with an increased production of thyroid antibodies and altered thyroid function, a combination of high TSH and thyroid autoimmunity in early pregnancy resulted in a 4-fold increase in the risk of GDM, as well as increasing the risk of adverse pregnancy outcomes (148). Anti-thyroid peroxidase (anti-TPO) has been detected in ~10–16 % of all pregnant women tested (146, 148) and in 80% of women who were screened specifically for GDM; however only 26% of all women who exhibited anti-TPO in their blood had a GDM diagnosis according to WHO criteria (149), suggesting that measurement of anti-TPO antibodies has low specificity for GDM screening.

Based on these observations it is likely that hormone fluctuations during pregnancy may play a role in the development of GDM and subsequent progression to T1DM.

##### Menopause

No significant differences were observed in age at menopause in women with T1DM, compared to controls in a 2011 Finnish study (150). These results contradicted previous studies reporting an earlier menopause age among women with T1DM, that claimed an average decrease in reproductive years by 17% (151). However, the Finnish study did note that patients with microvascular complications due to diabetes had a significantly earlier menopause (150). A meta-analysis (123), which included data from 200,000 participants, found a significant and clinically meaningful difference in the excess risk of mortality in female patients with T1DM compared to male patients, particularly in relation to mortality due to vascular causes. For macrovascular outcomes, such as cardiovascular and renal disease, the excess risk of mortality in women compared with men was even more prominent which could be due to the greater effect of hyperglycemia and diabetes seen in women than in men (152, 153).

In conclusion, young girls and women are more likely to be in a persistent state of poor glycemic control than young boys and men (154, 155), starting at puberty (124, 156, 157). An additional contributing factor is the disturbance in the hypothalamic-pituitary-ovarian axis in women, which triggers a chain of endocrinological events, starting with delayed menarche, menstrual irregularities and early menopause (158). As a result, even though the incidence and prevalence of T1DM in women may be equal to or lower than those reported among men, women bear an unequal burden of the disease and its sequelae throughout their lifetime, which is attributable to the endocrinological milieu unique to women.

#### Rheumatoid Arthritis (RA)

A study published in 1990 involving 564 patients with RA (159) reported an overall female to male incidence ratio of 2.3; however, with increasing age the female to male ratio decreased from 3.7 before 30 years of age to 1 after the 6th decade of life. The study also suggested that the average woman develops the first symptoms of RA at the time of menopause (159), with a peak age of RA onset around menopause (160). However, recent studies have suggested that overall prevalence of RA is four times higher in women than in men, and female to male incidence ratio of RA increases with age and is three to five times higher in reproductive and perimenopausal age group (161, 162) and in three regions in the world, namely, America, Europe and Western Pacific, over four out of five RA patients are women (162). Synovial fluid level of estrogens relative to androgens were found to be significantly elevated in both male and female patients with RA (163, 164).

##### Puberty

The polymorphism rs2476601 has been found to be significantly associated with juvenile RA in female but not in male patients, with evidence of a genotype-by-sex interaction (165). Early menarche at age <12 years was inversely associated with RA (166, 167) and found to be protective factor in one study (168). A study on an Egyptian population of boys and girls with juvenile rheumatoid arthritis found no significant difference in pubertal delay between male and female patients with RA in that country (169).

##### Pregnancy

Pregnancy and breastfeeding have been found to be a protective factor for RA (160, 170–172). The Swedish Nurses' Health Study, which was a 26-yearlong follow-up study with more than 120,000 female participants revealed that breastfeeding for >12 months was inversely associated with the development of RA, and demonstrated decreased risk of RA in postmenopausal women with a history of long-term breast feeding (173). The effect was dose-dependent and remained significant after adjustment for smoking and level of education (173). Irregular menstrual cycles and earlier age at menarche increased the risk of RA while other reproductive hormonal factors were not associated with an increased RA risk (173). A study on Chinese women in Guangzhou, China arrived at a similar conclusion that breastfeeding, especially of longer duration, but not oral contraceptive use, was positively associated with a lower risk of RA (174).

Possible explanations for the protective effect of breast feeding include long-term immunomodulation, such as the development of progesterone receptors on lymphocytes, dysregulated hypothalamic–pituitary–adrenal axis, and differences in cortisol concentrations. Lankarani-Fard et al. (175) measured cortisol concentrations in postmenopausal women, and noted significantly higher concentrations in those who had breast fed. In contrast, short-term breast-feeding may actually increase RA risk (176). Oxytocin, one of the hormones that is raised in women who breast feed, is known to reduce cortisol concentrations (177), induce well-being, and lower blood pressure in the mothers (178). Prolactin, which also is increased during breast feeding, is a known immunostimulator (179), and high concentrations of prolactin are seen in patients with RA (180, 181).

These observations suggest differential short-term and long-term effects of breast feeding on the immune system, and consequently on susceptibility to RA based on variable concentrations of different sex hormones before, during and after pregnancy.

##### Menopause

Early age at menopause (≤40–45 years) was found to be statistically significantly associated (182) with the subsequent development of rheumatoid factor (RF)-negative RA, whereas it was positively correlated with RF-positive RA, but the association did not reach statistical significance. The effect of early menopause on development of RA remained significant after adjusting for smoking, level of education, and length of breastfeeding (166, 183). The Canadian Early Arthritis Cohort Study on the other hand found that early age at menopause is significantly associated with seropositivity in women with early RA (184). An observational cohort study of RA patients enrolled in the Swiss Clinical Quality Management Program for Rheumatoid Arthritis, published in 2018, discovered that in women with RA, functional disability progression was less favorable in post-menopausal women compared to pre-menopausal women and was not explained by disease duration, age or radiographic damage (185) and a similar study in US women found that menopause is associated with a worsening progression of functional decline (186). A cohort study focusing on pathological joint damage found that although patients >60 years of age of both sexes suffering from RA had greater joint damage compared to younger patients (both male and female), older postmenopausal female patients had most severe disease in terms of joint destruction and physical disability (187). The study concluded that the menopausal state is responsible for the major part of the differences in outcome between men and women in RA (187). This conclusion is also supported by a population-based control study (188) demonstrating the protective effect of post-menopausal hormone therapy in RA.

#### Psoriasis

Prominent increase in incidence of psoriasis is observed in the sixth decade of life which corresponds to the postmenopausal period (189–191). Another set of studies found a bimodal distribution of ages of onset for psoriasis; puberty and between the ages of 30 and 50 years (192–194).

##### Puberty

The peripubertal increase in the prevalence of psoriasis may be explained by the increase in sex hormones during this period since sex hormones are known to promote keratinocyte differentiation (195). High levels of estrogens seem to have a regulatory and inhibiting effect on many components of the immune response, while low levels can be stimulating (106, 196, 197) and similar to other T cell mediated autoimmune diseases, such as MS and RA, estrogen is protective in psoriasis.

##### Pregnancy

Psoriatic body surface area (BSA) was found to significantly decrease between the 10th and the 20th week of gestation compared to that in controls, while BSA significantly increased by 6 weeks postpartum (198) suggesting protective role of pregnancy in psoriasis. Pregnancy's natural immunomodulation is associated with alleviation of symptoms in patients suffering from psoriasis (199, 200). A number of studies have investigated the association between hormones and psoriasis (201–203). A worsening of psoriatic symptoms has been observed postpartum, prior to menses, and at menopause, concomitant with decrease in estrogen and progesterone levels, while most patients undergoing hormone therapy around menopause noted no change in their symptoms. Although progesterone levels alone did not correlate with changes in psoriatic symptoms among pregnant women, a higher ratio of estrogen to progesterone resulted in improvement in symptoms in a group of 47 patients (198).

##### Menopause

A decrease in estrogen level during menopause was reported to affect the occurrence or exacerbation of psoriasis in patients already suffering from the condition, and it has been postulated that reduced estrogen levels lead to insufficient inhibition of Th1 cell-mediated responses in menopausal women and consequent disease exacerbation (204).

### Mechanistic Perspective: Hormones, Transition States, and Epigenetics

MS, RA, Psoriasis and T1DM are considered to be Th1 mediated autoimmune diseases while in case of SLE, Th2-mediated autoimmunity is believed to predominate. However, at least one study found a Th2-skewed immune response in adult patients with T1DM (205).

#### Endocrine Transition and Autoimmunity: Reproductive Hormones

T-cell-mediated autoimmunity is upregulated post-puberty, as demonstrated by Ahn et al. (52) who observed that female post-pubertal mice developed enhanced myelin-reactive T-cell responses, compared to age-matched mice that had been prevented from entering puberty via pre-pubertal ovariectomy. Similarly, Makino et al. (206) demonstrated a reduced incidence of type 1 diabetes mellitus (T1DM) with pre-pubertal ovariectomy in non-obese female diabetic mice, whereas pre-pubertal castration in male mice increased the risk of T1DM suggesting inherent protection enjoyed by the male sex. A study also demonstrated hormone-dependent gender-specific splenic immune response post-puberty where female mice exhibited higher expression of adaptive immune response genes while male mice had higher innate immune response genes' expression (207). A significant gender-dependent divergence in serum immunoglobulins levels was also noted in the study (207). No statistically significant pre-pubertal differences were noted in this study.

Estrogen Receptor—alpha is expressed in pancreatic beta cells and sex hormones also exert effects on beta cell function (208). Exogenous estrogen might limit islet amyloid polypeptide-mediated beta cell loss in mice (209). Even though increased prevalence and severity of islet amyloid deposition has been identified in males compared to females with Type 2 Diabetes Mellitus (T2DM) (210), in T1DM, beta cell destruction was increased in females compared to males after puberty (211), potentially due to the influence of sex hormones on the immune system in the immune-mediated T1DM. Puberty may accelerate onset of T1DM in genetically susceptible females, mediated by the effect of estrogen on the Interleukin-6 (IL6) promoter (212). Anti-islet autoantibodies have been detected years before clinical diagnosis of T1DM (213) and these antibodies, which play an important role in T1DM disease development, are more frequently inherited paternally than maternally, even though frequencies of these autoantibodies were found equal in male and female offspring (214).

During peripubertal thymic involution, androgens stimulate CD8+ cells and reduce the CD4+/CD8+ ratio, which diminishes cell-mediated immune responses in male mice and rats, while estrogen has the opposite effect, supporting CD4+ T cell survival (215, 216). Compared to healthy women serum testosterone was reduced in women suffering from Th1-mediated autoimmune disease such as MS, particularly during the active phase of the disease, as documented by brain MRI, while no significant difference was seen in sex hormone levels between men suffering from MS and healthy men (217). Estrogen may be both pro- (218) and anti-inflammatory (219), depending on the circulating levels in the blood as well as cell-specific receptor activation (49, 220). High-estrogen states seem to favor amelioration of symptoms in T-cell mediated autoimmune disorders such as MS and RA, while a low-estrogen state is associated with disease progression (221).

Important role of estrogens in SLE pathogenesis has been long-suspected but the molecular mechanisms involved remain to be definitively elucidated (222, 223); however recent evidence suggests that rapid turnover of ER-alpha receptor molecules in T cells from SLE patients due to cellular level alterations in the ubiquitination signaling pathway may be responsible (224).

Estrogen was also found to inhibit the production of IL-12 and TNF-alpha (203), suppress antigen-presenting capacity in dendritic cells, and normalize type 1-shifted T cell priming by dendritic cells (225) as well as stimulate anti-inflammatory IL-10 production in dendritic cells and T cells (226), thereby conferring protective effect on women in the reproductive age group against Th1-mediated psoriasis.

Both early (four weeks of age) and late (12 weeks of age) estrogen administration protected non-obese diabetic (NOD) mice from spontaneous autoimmune diabetes up to 30 weeks of age via revival of invariant natural killer T (iNKT) cells' immunomodulatory function (227). Early estrogen administration averts insulitis that would signal loss on pancreatic beta cells and delayed treatment ameliorates insulitis to thwart the destruction of inflamed islets through what the authors described as “bystander effect” (227).

Hormonal fluctuations in pregnancy (228) and the associated exacerbations of SLE are well-documented (229, 230). Estrogen has been traditionally associated with SLE (231, 232). At plasma levels experienced during pregnancy, estrogen inhibits Th1-mediated pathways, through mediators such as interleukin-1 (IL-1), interleukin 6 (IL-6), tumor necrosis factor-alpha (TNF-α), suppresses the activity of natural killer (NK) cells, and stimulates Th2-mediated pathways, through mediators such as interleunkin-4 (IL-4), interleukin-10 (IL-10), and Transforming Growth Factor—beta (TGFβ) (106), as well as enhances the number and function of CD4+ CD25+ regulatory T cells (233, 234). At lower concentrations than those observed during pregnancy, estrogen stimulates release of Th2-response mediating cytokines, promotes NK cell activity (106) and stimulates antibody production by B cells (106). In fact, a 1-year pilot study (235) in 16 patients demonstrated that blocking estrogen receptors *in vivo* by an estrogen selective receptor downregulator could be considered as a new and relatively safe therapeutic approach in the management of SLE patients with moderately active disease.

During the perimenopausal transition, declining levels of estrogen and dehydroepiandrosterone sulfate may be associated with increased production of Th1 cytokines such as IL-1, IL-6, TNF-α, and increased response to these cytokines, decreased secretion of Th2 cytokines, decreased lymphocyte levels (CD4+ T cells, B cells), and decreased cytotoxic activity of NK cells (236).

Based on above observations, it is clear that hormones significantly affect the immune system (86) and there is strong evidence that estrogens have immunomodulatory effects (237–239). The role of hormone replacement therapy and estrogen receptor modulators in autoimmune diseases is being explored (240–246).

Thyroid autoimmunity has been described as a “window” into autoimmune states and has been covered in multiple reviews (247–249). Individuals suffering from more than one autoimmune disease are likely to have a co-existing thyroid autoimmune state as well, which may have been present in either clinical or subclinical form since first diagnosis of another autoimmune disease (248). It is possible that hormonal flux in susceptible women may trigger or precipitate downstream changes that disturb the fragile balance between inflammation and immune regulation, similar to a neurological “tipping point” described in perimenopause that results in hypometabolism, insomnia, depression and ultimately neurological decline (250).

#### Endocrine Transition and Autoimmunity: Other Factors

Leptin has been implicated as another hormone potentially responsible for the sexual dimorphism in post-puberty autoimmune diseases (251). Leptin is necessary for the induction of MS in in leptin-deficient, C57BL/6J-ob/ob mice (252). Leptin levels continue to rise in post-pubertal females, but not in males due to the suppressive effect of testosterone on leptin secretion (253). Furthermore, injection of recombinant leptin in male mice increases their susceptibility to developing experimental autoimmune encephalomyelitis (254). Obesity and therefore leptin are implicated as central triggers of unnecessary or aggressive inflammatory state responsible for autoimmune states and the increased incidence of autoimmunity could be a function of increased leptin, while in men testosterone acts as an immunosuppressant. This hypothesis is lent credence by a study in patients with Hashimoto's thyroiditis (both hypothyroid and euthyroid) where body mass index and fat mass was higher in patients compared to controls (255).

Prolactin is another pro-inflammatory hormone implicated in development of autoimmune diseases due to its increased concentrations found in post-pubertal females compared to men (179). Significantly higher prevalence of autoimmune thyroid diseases was found in female prolactinoma patients compared to age-matched healthy women (256). Similarly, SLE patients had higher leptin levels compared to controls and these levels were correlated with disease activity and severity (257). Increased leptin in SLE also showed an inverse correlation with the frequency of T_reg_ cells (257).

Not all autoimmune pathogenesis can be attributed to hormonal influence. Etiopathogenesis of Th2-mediated autoimmune diseases such as SLE has been attributed to the sexual dimorphism of the immune response, initiated in the gut mucosa (258). Female mice were found to have higher plasma cell- and gut-imprinted-α4β7 T cell frequencies, markedly higher CD45+ immune cell densities, and higher numbers of IL-17-, IL-22-, and IL-9-producing cells in the lamina propria compared to male counterparts (258).

Prepubertal pediatric autoimmune diseases such as Juvenile Rheumatic Arthritis peak between the ages of two and four when levels of both estrogen and testosterone are low (259) and direct hormonal influence on autoimmunity is likely minimal. *In utero* sex steroid levels are much higher than in childhood but reach low levels after birth, but approximately around the 6-month mark estrogen and testosterone levels reach between one-fifth to one-third of adult levels in female and male children, respectively, and this period has been termed “mini-puberty” (260). It is possible that this rise in levels of sex hormones soon after birth primes genetically susceptible individuals to develop autoimmune diseases in early childhood or later in life. Epigenetic mechanisms, discussed later, could also play a role in prepubertal autoimmune diseases (261–265).

Pregnancy results in a shift from a pro-inflammatory and cell-mediated (Th1) type of immune response to an anti-inflammatory and antibody-mediated (Th2) type of immune response, which promotes fetal survival due to diminished Th1 responses involved in rejection of the fetus as an allograft (266). After pregnancy, Th1 immune-mediated diseases reappear (267–270). Pregnancy has little effect on long-term disability in women suffering from MS according to some sources (32, 271), although one study found pregnancy and childbirth associated with less long-term disability (272). Breastfeeding is protective in Th1 mediated disease and women with MS who breastfed were found to have an almost 50% lower risk of post-partum relapse (273) compared to women who did not breastfeed. Although breastfeeding seemed to protect women from relapse, a considerable body of literature covered in an excellent review (274) implicates prolactin as one of the causes of the post-partum surge in MS symptoms, and this phenomenon is similar to higher prolactin levels found in post-pubertal female subjects that plays a role in increasing their susceptibility to Th1-mediated autoimmune diseases (179). This contrasting phenomenon can be explained by the observation that prolonged breastfeeding was found to decrease proinflammatory CD4+ tumor necrosis factor-α-producing cells in both healthy women and women with MS, but cell counts increased again after menses resumed (275).

With respect to gestational diabetes, changes in concentrations of two chemokines—an increase in level of the chemokine Monocyte Chemotactic Peptide (MCP)-1 levels, and decrease in levels of another chemokine, RANTES (Regulated on Activation Normal T-cell Expressed and Secreted)—is implicated in the pathogenesis (276). MCP-1 is a pro-inflammatory activator of several immune cells (277), and RANTES is an immunomodulator suppressing the maternal allogeneic response (278). In diabetic pregnancies, increase in MCP-1 and decrease in RANTES will elevate pro-inflammatory response and attenuate the immunosuppressive effect of RANTES.

#### Endocrine Transition and Autoimmunity: Influence of Epigenetics

Recently it has come to light that the autoimmune regulator (AIRE) is key to central tolerance of self-antigens and hormonal action affects the expression of AIRE mRNA and protein (279). Estrogen as well as male castration downregulated AIRE, while estrogen-mediated methylation of CpG sites in the promotor region of AIRE could potentially disturb the delicate balance between autoreactive T cells and T_reg_ cells, precipitating clinical autoimmune disease(s) in the presence of environmental trigger(s) (279). The role of epigenetics and DNA methylation was also explored by our lab and we found that DNA methylation plays a key role in progression toward reproductive senescence (280), which could have crucial implications for autoimmune states observed in post-menopausal women. Inhibition of DNA methylation expedited transition to reproductive senescence in female Sprague-Dawley rats, while increased methylation through methionine supplementation prolonged the period of endocrine aging by preserving regular cycling (280). Another theory suggests that environmental toxins termed “endocrine disruptors” play a key role in the increased incidence of autoimmune diseases, cancer and diabetes by altering the genome and the epigenome (281). Age related immune dysfunction in innate and adaptive immune system regulated via epigenetic mechanisms has been observed (282) and implicated in autoimmune disease states. Epigenetically induced immunosenescence potentially leads to elevated levels of proinflammatory cytokines during the aging process either due to accumulated toxins or the normal aging process or both and consequently increases the susceptibility to autoimmune diseases in aging (283). These age-related changes occur in both sexes but testosterone's immunosuppressant function and its decline much later in life (284) compared to estrogen's varying effects on the immune system, hormonal flux in women and earlier loss due to menopause amplify the effects of immune system-related changes in females compared to males. Epigenetic mechanisms of MS (285), SLE (286, 287), RA (288), T1DM (289) and psoriasis (290) has been studied in some detail but further elucidation is necessary to fully understand the role of epigenetics as a driver of menopausal transition and autoimmunity in females.

Similar to menopause, puberty too has a strong epigenetic component as articulated by Toro et al. (291), and epigenetic mechanisms are likely a bridge between external non-genetic stimuli (environment, nutrition, physical activity) and genomic expression or repression that serves to modulate puberty. In fact, Thompson et al. (292) discovered that in females, puberty associated DNA methylation changes at CpGs are in close proximity to estrogen responsive genes and form networks centered on respiratory and inflammatory processes. Moreover, the authors suggest that these epigenetic changes that materialize during puberty in females likely contribute to the sexual dimorphism of immune-mediated diseases later in life (292). Some of these epigenetic mechanisms could be DNA methylation, histone post-translational modifications and non-coding RNAs (291) that have been known to influence various autoimmune diseases (293, 294). To add to this complexity, Markle et al. (295) showed that microbial exposure in early-life affects level of sex hormones and influences autoimmune states in non-obese diabetic mice via sex hormone regulation. Male NOD mice were conferred protection against T1DM by gut microbiota and this protection was transferable to immature female NOD mice via transfer of microbiota, which increased testosterone in the female mice, reduced islet cell inflammation and decreased antibody production (295).

Based on these observations, the epigenetic regulation of perimenopausal and peripubertal transition states could be the missing link that connects hormonal flux, genetic susceptibility and environmental stimuli in the initiation, pathogenesis and clinical manifestation of autoimmune diseases. Future studies can shed light on the exact molecular pathways as well as clarify causal relationships between the different factors that ultimately cause autoimmune disease states to manifest.

## Conclusion

**Figure 2:**
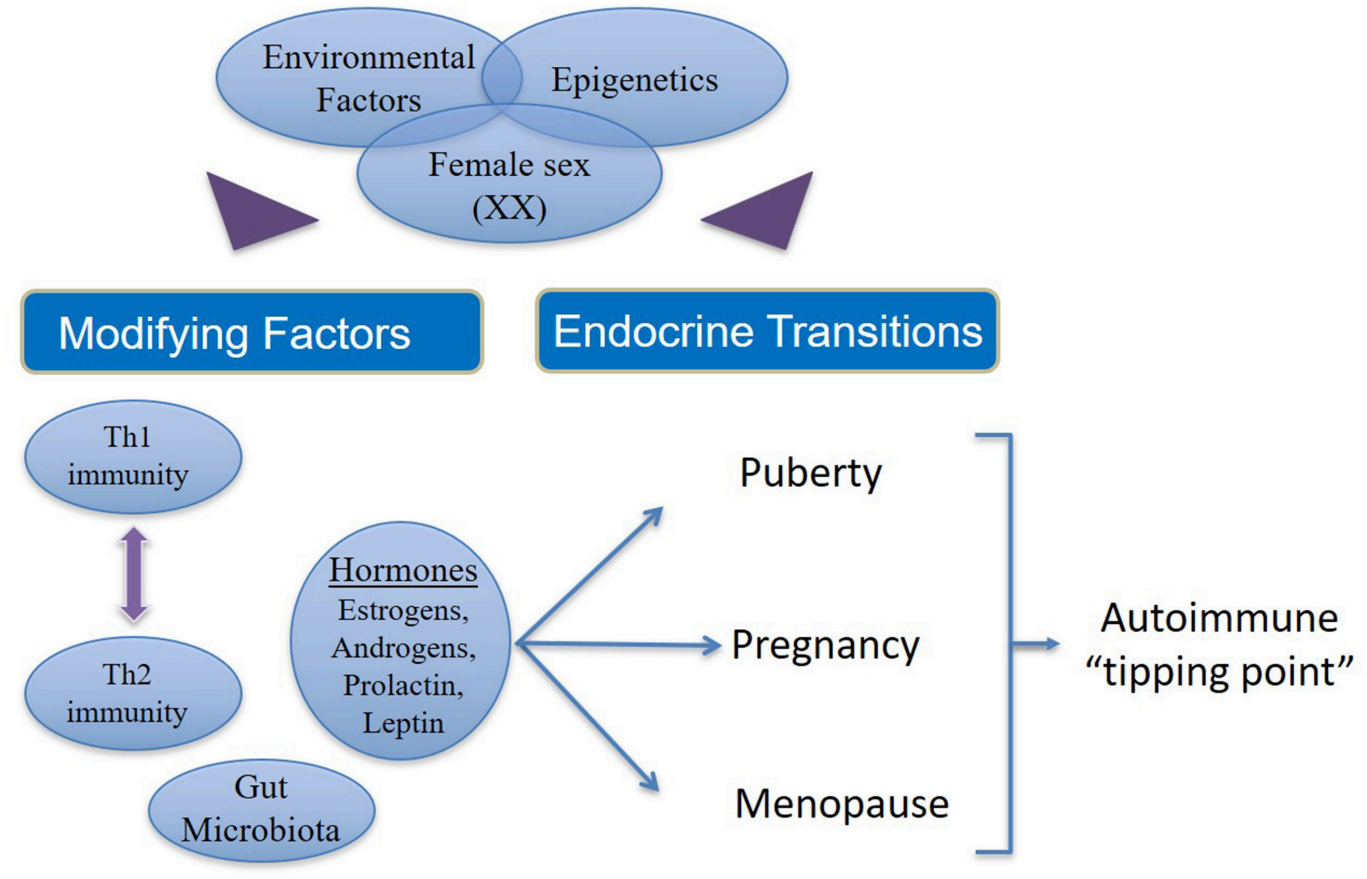
Factors that contribute to increased incidence and prevalence of autoimmunity in women. In women (46XX) with genetic susceptibility to autoimmune states, external environmental stimuli affect modifying factors as well as endocrine transitions via epigenetic mechanisms. Additionally, there are interactions between estrogens, androgens, leptin and prolactin on one hand and the interplay between Th1 and Th2 immune responses on the other. Both (endocrine and immune response) these phenomena are influenced in varying ways during the female transition states depending on the circulating concentrations of different hormones and immune cytokines, which in turn may be determined by epigenetics. Thus, hormonal fluctuation, immune polarization and transition states together drive susceptible women over the autoimmune “tipping point” leading to manifestation of overt clinical disease.

Autoimmune disease states show strong associations with endocrinological changes in human and animal studies. There is clear evidence for the role of sex steroids in the immune disturbances that result in autoimmune diseases (Figure 2). The majority of women who pass through the different endocrinological transition states do not succumb to autoimmune diseases.

However, a small percentage of women emerge with an increased risk of developing autoimmune diseases due to sustained hormonal changes during the endocrinological transitions coupled with genetic susceptibility and environmental injury, which are likely modulated by epigenetic mechanisms. In women there are interactions between estrogens, androgens, leptin and prolactin on one hand and the interplay between Th1 and Th2 immune responses on the other. Both (endocrine hormones and immune responses) these phenomena are influenced in varying ways during the female transition states depending on the concentration of different hormones and immune cytokines. Thus, hormonal fluctuation, immune polarization, and transition states together increase the susceptibility of women to autoimmune diseases.

**Figure 3:**
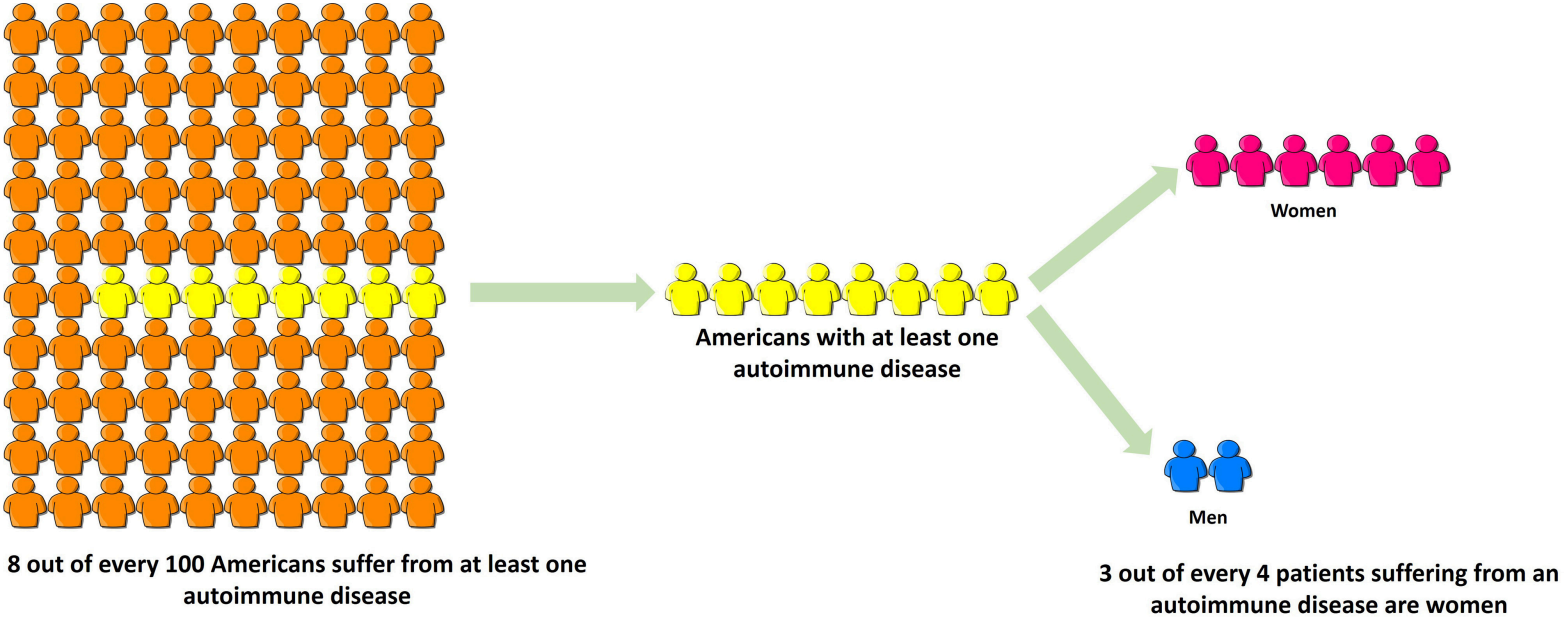
Three-fourths of all patients suffering from autoimmune disease are women. Based on the source of information, it is estimated that 5–8% (NIH, 2005) to 20% (American Autoimmune Related Diseases Association, 2017) of all Americans suffer from at least one autoimmune disease, of which ~78% or three-fourths patients are female, and the rest are male. Despite this, autoimmune diseases are rarely discussed as a women's health issue.

Autoimmune diseases are highly debilitating diseases with no cure and only moderately satisfactory but expensive treatment that nonetheless increases patients' vulnerability to deadly infections due to prolonged immunosuppression. Autoimmune diseases result in considerable erosion in quality of life, unemployment or underemployment and increased caregiver hours. Based on the source of information, it is estimated that 5–8% (1) to 20% (American Autoimmune Related Diseases Association; https://www.aarda.org/knowledge-base/many-americans-autoimmune-disease/; accessed on Nov. 20, 2018) of all Americans suffer from at least one autoimmune disease, of which ~78% or three-fourths patients are female, and the rest are male (Figure 3) (296). Despite this, autoimmune diseases are rarely discussed as a women's health issue. The incidence and prevalence rates of various autoimmune diseases are rising all over the world (297, 298). At the global level, increased incidence and prevalence of autoimmune diseases in Western and Northern countries compared to Southern and Eastern countries has led to speculation that alterations in dietary habits such as highly prevalent Western diet, increased exposure to pollution as well as a changing environment may be responsible for this region-specific rise (297). The National Institutes of Allergy and Infectious Diseases (NIAID) in 2011 estimated that the cost of treating autoimmune disease in the US is >$100 billion annually (299); this excludes indirect costs to the patient and family members incurred due to decreased quality of life and loss of productivity. In contrast, autoimmune diseases research funding from NIH was $883 million in FY 2016 and $821 million in FY 2015 (300). Recently women's health issues have received more attention, and considering autoimmune diseases are a leading cause of death among young and middle-aged women in the United States (12), the plight of autoimmune disease patients should not go unnoticed. Increased funding for research in autoimmune diseases and exploring their link to endocrine transitions, raising awareness among healthcare providers and the general population and developing better support systems for both men and women suffering from autoimmune diseases are some ways to mitigate the toll autoimmune diseases take on our society.

Greater understanding of: (1) the underlying cellular and molecular level immune changes due to endocrinological transitions; (2) the genetic and epigenetic characteristics of women who have increased likelihood of developing autoimmune diseases; and the (3) translational animal models currently used to study endocrinological transition states in women could help predict, potentially prevent and even cure the debilitating group of autoimmune diseases in women.

## Author Contributions

All authors listed have made a substantial, direct and intellectual contribution to the work, and approved it for publication.

### Conflict of Interest Statement

The authors declare that the research was conducted in the absence of any commercial or financial relationships that could be construed as a potential conflict of interest.
